# Effects of the Baking Process on the Chemical Composition, Sensory Quality, and Bioactivity of *Tieguanyin* Oolong Tea

**DOI:** 10.3389/fnut.2022.881865

**Published:** 2022-05-16

**Authors:** Ying Gao, Qing-Qing Cao, Yu-Hong Chen, Daniel Granato, Jie-Qiong Wang, Jun-Feng Yin, Xue-Bo Zhang, Fang Wang, Jian-Xin Chen, Yong-Quan Xu

**Affiliations:** ^1^Tea Research Institute, Chinese Academy of Agricultural Sciences, National Engineering Research Center for Tea Processing, Key Laboratory of Tea Biology and Resources Utilization, Hangzhou, China; ^2^Department of Biological Sciences, Faculty of Science and Engineering, University of Limerick, Limerick, Ireland; ^3^National Tea Quality Supervision and Inspection Center, Fujian, China

**Keywords:** oolong tea, baking process, free radical scavenging activity, anti-advanced glycation end products, antibacterial, sensory properties, catechins

## Abstract

*Tieguanyin* oolong tea (TOT), a semi-oxidized tea originating from Anxi county in China, is categorized into jade TOT, medium-baked TOT, and deep-baked TOT, based on different baking processes. To study the effects of baking, chemical analysis, sensory evaluation, and bioactivity assessments of the three TOTs were conducted. The results indicated that the baking process promoted the formation of colored macromolecules (e.g., theabrownins), which affected the color of tea infusion. Free amino acids underwent the Maillard reaction and generated specific Maillard reaction products, such as 5-hydroxymethylfurfural and furfural, which modified the taste and aroma. Floral and fresh volatiles were remarkably reduced, while multiple new volatiles were produced, forming a typically baked aroma. The antioxidant activity and antibacterial activity were reduced after baking, which might be associated with the decrease of monomeric catechins. These results provide a scientific basis for understanding the changes caused by the baking process.

## Introduction

*Tieguanyin* tea (TOT) is a semi-oxidized oolong tea from Anxi county, Fujian province, China. It is popular in south China and among Chinese expatriates in Southeast Asia. Jade TOT, medium-baked TOT, and deep-baked TOT are three significant types of TOTs. The jade TOT is produced by harvesting fresh tea leaves, withering, bruising, partial oxidation, fixing, shaping, and drying (1). The medium-baked TOT is produced by baking the jade TOT, and the deep-baked TOT is produced by baking the medium-baked TOT once more. The first baking is usually set at about 105°C for 3–8 h, and the second baking is set at about 115°C for 2–6 h. The three TOTs have distinguishing flavor characteristics and different target customers. The jade TOT is green, smells floral, tastes brisk and is preferred by females and youngsters (2). The deep-baked TOT is the favorite for the locals in Anxi county and is the most traditional and expensive TOT. Compared with the jade version, the deep-baked TOT color is darker, the aroma and the taste are more complex. The deep-baked TOT looks brownish, has a typical roast aroma, tastes mellow and thick with a strong sweet aftertaste. The sensory property of the medium-baked TOT falls in between the above two TOTs. Many customers feel that the flavor of medium-baked TOT is mediocre, not as distinctive as the other two TOTs. Therefore, medium-baked TOT is the least popular one among the three TOTs.

The flavor is caused by a specific combination of taste and aroma compounds. As a semi-oxidized tea, TOT not only contains flavor components originally from fresh tea leaves, but also flavor components generated during processes, especially the partial oxidation process. Plenty of them is oxidized intermediates, which may further convert to other substances under heat treatment or long-term storage (3), leading to the flavor alteration of TOT. Catechins are one of the flavor components which are remarkably changed during oolong tea processing. Catechins, featuring secondary metabolites in tea, are vital contributors to tea infusions’ bitter and astringent taste. However, they are chemically active, and part of them may undergo oxidation and polymerization to form new flavor molecules like theaflavins (TFs) and theasinensins during oolong tea processing (4). These intermediates can continue to form complex molecules under certain conditions (5). As catechins and their derivatives possess different sensory properties, the changes in these chemicals affect the sensory profile of the tea. Wang et al. found that by baking green tea, the composition and content of catechins were altered, resulting in a less astringent taste (6).

It is worth noting that many flavor compounds in tea are also bioactive compounds, suggesting that the alteration of bioactivity may occur along with the flavor alteration of tea. For example, catechins, mentioned above as major taste components, are the main bioactive components for the antioxidant and antibacterial activity of unfermented and semi-fermented tea (7). Lv et al. demonstrated that the content and composition of catechins in green teas made with different enzyme-inactivating processing technologies were varied, and the chemical composition was correlated to the sensory property and the antioxidant activity of green teas (8). Wang et al. proved that the baking process modified the sensory quality of Wuyi rock tea, as well as decreased the total phenolic content and free radical scavenging activity (9).

The baking process to produce medium-baked and deep-baked TOTs is conducted in a heated environment where the temperature exceeds over 100°C. Under this condition, not only catechins, but also other vulnerable components tend to transform into more stable products. To find out how baking dramatically converts the flavor and whether baking modifies the bioactivity, the differences among jade TOT, medium-baked TOT, and deep-baked TOT on the chemical composition, sensory quality, free radical scavenging activity, anti-advanced glycation end products (AGEs) activity, and antibacterial potential were investigated.

## Materials and Methods

### Tea Samples

The jade TOT (BT0), medium-baked TOT (BT1), and deep-baked TOT (BT2) samples were provided by Chanxinyuan (Fujian) Tea Industry Co., Ltd. (Fujian, China). BT0 was baked at 107°C for 210 min after 12-day storage to produce BT1 and then baked at 115°C for 150 min after 22-day storage to produce BT2.

### Preparation of Infusions

Each tea sample was ground and filtered through a 60 Tyler mesh sieve. The tea powder was brewed with distilled water (3:500 w/v) at 100°C for 40 min, cooled to room temperature, and centrifuged at 8,000 *g* for 10 min to obtain the supernatant for the analysis of non-volatile chemical composition. Part of the supernatant was vacuum dried to prepare the tea extracts to assess antibacterial activity. The detailed parameters of vacuum drying were frozen at −30°C for 3 h and freeze-dried for 36 h (0–33 h increasing to 25°C and kept at 25°C for 3 h). The vacuum level was less than 50 Pa.

### Sensory Evaluation and Instrumental Color

Based on the national standard GB/T 23776-2018 (10), the color, aroma, and taste of the three tea samples were evaluated by a professional sensory evaluation team consisting of seven qualified panelists, with ages ranging from 25 to 50 years old, three males and four females. The intensities of aroma and taste attributes were scored. Score 0–2 mean “extremely weak,” 2–4 mean “weak,” 4–6 mean “neutral,” 6–8 mean “strong,” and 8–10 mean “extremely strong.” Each evaluation was replicated three times on different days with a randomized order of samples for each test to assure reproducibility in the sensory analysis.

The color analysis of tea infusions was measured by a spectrophotometer (Konica Minolta, CM-3500d) by recording the CIE L*a*b* color space parameters.

To determine which part of the tea infusion contributed to the differential colors among samples, Vivaspin 20 ultrafiltration units (Product Nos. 28932358, 28932360, and 28932362, Cytiva, Marlborough, MA, United States) were used. Each tea infusion was filtered through membranes with a 3/10/50 kDa molecular weight cut-off, accordingly, *via* centrifuging at 4,000 *g* for 40 min at 37°C.

### Determination of Non-volatile Chemical Composition

The total phenolic content, free amino acids, soluble proteins, soluble sugars, soluble polysaccharides, flavones, TFs, thearubigins (TRs), and theabrownins (TBs) were measured according to previously published methods (11). In brief, the contents of total polyphenols and free amino acids were determined based on the national standard GB/T 8313-2008 and GB/T 8314-2013, respectively (11). The content of soluble proteins was determined using a commercial protein assay kit (Bradford Protein Assay Kit, Product No. P0006, Beyotime Biotechnology, Haimen, China). The content of flavones was determined according to the following procedures. A 0.5 mL sample solution was added to 10 mL 1% aluminum trichloride, mixed, stayed at room temperature for 10 min, and read the absorbance at 420 nm. The contents of TFs, TRs, and TBs were determined by Robert’s method (12). The contents of eight catechins, gallic acid, and caffeine were determined using an HPLC method (13).

The content of soluble sugars was determined using the anthrone-sulfuric acid method. One milliliter sample solution was added to 4 mL anthrone-sulfuric acid (2 mg/mL), mixed, water-bathed at 100°C for 10 min, cooled to room temperature, and read the absorbance at 620 nm. The determination of soluble polysaccharides was the same as that of soluble sugars but with different pretreatment. The sample solution was added to 95% ethanol (1:5 v/v), stored at 4°C overnight, centrifuged at 8,500 *g* for 10 min to get the polysaccharide precipitation, and re-dissolve it with distilled water to prepare the solution for the anthrone-sulfuric acid assay. The contents of free amino acid components were determined using an amino acid analyzer (Hitachi 835-50, Tokyo, Japan) with a former established method (14).

The untargeted analysis was carried out using a previously established UPLC-QE-Orbitrap-MS method (11). The separation was performed on an ACQUITY UPLC HSS T3 column (1.8 μm, 2.1 mm × 100 mm, Waters, Milford, MA, United States) using a Dionex Ultimate 3000 RS system (Thermo Fisher). A 0.1% formic acid in water and acetonitrile was used as mobile phases A and B. The gradient changes of mobile phases were 0–1 min, 5% B; 1–2 min, 5–10% B; 2–6 min, 10–35% B; 6–8.5 min, 35–100% B; 8.5–9.5 min, 100% B; 9.5–10 min, 100–5% B; and 10–12 min, 5% B. The total flow rate was 0.3 mL/min. The column temperature was 40°C. The MS analysis was conducted using the QE-Orbitrap mass spectrometer (Thermo Scientific, United States) with electrospray ionization (ESI), operating in the positive and negative ionization full scan modes. Auxiliary gas and sheath gas flow rates were 10 and 45 (arbitrary units), respectively. The auxiliary gas heater temperature was 300°C. The capillary temperature was 320°C. The spray voltage was 3.1 kV and the S-lens RF level was 50 V. The normalized collision energy (NCE) was 30 eV. The resolution of the full scan and ddMS2 were 70,000 and 35,000, respectively. The full MS scan ranges were set from *m/z* 66.7 to 1,000. Data were acquired and processed using ThermoXcalibur 3.0 software (Thermo Scientific, United States). Tentative identification of non-volatiles was based on comparing retention time, *m/z* values, and MS/MS fragments with standards or data from databases (e.g., Massbank and MzCloud) when standards were unavailable. Relative quantitation was calculated by comparing the relative intensities of the parent ions among samples and presented in a heat map after converting to *Z*-scores of the rows.

### Determination of Volatile Chemical Composition

The volatile chemical composition was investigated by the headspace solid-phase micro-extraction/gas chromatography-mass spectrometry (HS-SPME-GC-MS) (15). Before the extraction, the fiber of the SPME needle [50/30 μm divinylbenzene/carboxen/polydimethylsiloxane, StableFlex (2 cm), Product No. 57348-U, Supelco, Bellefonte, PA, United States] was kept at 250°C for 10 min to remove the remaining volatiles. A 0.5 g tea powder was added to a 50 mL glass vial and mixed with 5 mL boiling water and 30 μL ethyl caprate (internal standard). The glass vial was sealed immediately, gently vortexed, and incubated at 60°C. An SPME needle was inserted into the glass vial through the cap to absorb volatiles for 1 h. Then, the SPME needle was inserted into the injection port of GC to desorb volatiles at 250°C for 5 min.

Volatile organic compounds were determined by an Agilent 6890 gas chromatograph coupled with an Agilent HP 5973 mass selective detector (Agilent, Wilmington, DE, United States). The separation was performed on a DB-5MS capillary column (30 m × 250 μm × 0.25 μm) with the following GC conditions, which were the GC inlet temperature of 250°C, the split ratio of 15:1, the carrier gas (high purity helium) flow of 1.0 mL/min, the linear flow velocity of carrier gas of 40 cm/s. The column temperature was set as follows: 0–2 min, 40°C; 2–24.5 min, 40–85°C; 24.5–26.5 min, 85°C; 26.5–64.5 min, 85–180°C; 64.5–66.5 min, 180°C; 66.5–71.5 min, 180–230°C; and 71.5–73.5 min, 230°C.

The MS conditions were the temperature of the ion source of 230°C, the voltage of 70 eV, and the scan ranging from *m/z* 40 to 400. Tentative identification of volatiles was made by comparing the MS fragmentation patterns with data from the National Institute for Standards and Technology database (NIST 08, match percentage >80%). The relative abundance of each volatile was calculated by comparing the peak area of each compound to the total peak area.

### Determination of *in vitro* Antioxidant Activity

The 2,2-diphenyl-1-picrylhydrazyl (DPPH) radical scavenging activity was assessed using the protocol described by Xu et al. (16). The 2,2′-azinobis-(3-ethylbenzothiazoline-6-sulfonic acid) (ABTS) scavenging activity was determined according to the protocol described by Re et al. (17). The hydroxyl radical scavenging activity was assessed using a commercial kit (Hydroxyl Free Radical Assay Kit, Product No. A018-1-1, Nanjing Jiancheng Bioengineering Institute, Nanjing, China), according to the manufacturer’s instructions. All analyses were conducted in triplicates.

### Determination of Anti-advanced Glycation End Products Activity

The effects of TOTs on the formation of AGEs were investigated in bovine serum albumin (BSA) + glucose, BSA + methylglyoxal, and BSA + glyoxal systems, respectively, based on a previously published method with some modifications (18). In the BSA + glucose system, 200 μL of 15 mmol/L glucose, 200 μL of 30 mg/mL BSA, and 200 μL of tea infusion were mixed and incubated at 100°C for 1 h. The relative fluorescence was measured using a multi-functional microplate reader (excitation/emission = 370/440 nm) (Thermo Scientific Varioskan Flash, Waltham, MA, United States). In the BSA + methylglyoxal system, glucose was replaced by 200 μL of 1.5 mmol/L methylglyoxal. In the BSA + glyoxal system, glucose was replaced by 200 μL of 1.5 mmol/L glyoxal.

### Determination of Antibacterial Activity

The minimum inhibitory concentrations (MICs) against *Salmonella typhi* [CMCC (B) 50071], *Shigella flexneri* [CMCC (B) 51572], β-hemolytic *Streptococcus* [CMCC (B) 32210], *Staphylococcus aureus* [CMCC (B) 26003], and *Escherichia coli* [CMCC (B) 44102] were determined using the micro-dilution method (19).

Each strain was plated on an agar plate and incubated at 37°C for 24 h. A single colony was used to inoculate 10 mL of sterile broth and incubated at 37°C for 24 h. Then the suspension was diluted to 2 × 10^5^ CFU/mL. To investigate the effects of the three tea samples on the growth of each strain, BT0, BT1, and BT2 extracts were dissolved in sterile broth to prepare a 2 mg/mL solution and serially diluted to reach the final concentrations of 0.5, 1, and 2 mg/mL, respectively. A 100 μL bacterial suspension was mixed with 100 μL tea extract solution and then added to a 96-well plate. The positive control was 100 μL bacterial suspension mixed with 100 μL sterile broth. The 96-well plate was incubated overnight at 37°C and then observed. MICs are defined as the lowest concentration of an antimicrobial agent that would inhibit the visible growth of a microorganism after overnight incubation.

### Statistical Analysis

The data are presented as the mean ± standard error of the mean (SEM). All experiments were carried out in triplicate and repeated in three independent sets of experiments. The results were analyzed with SPSS version 18.0 for Windows (SPSS, Chicago, IL, United States), using a one-way analysis of variance and a *post hoc* test (two-sided Dunnett’s test) to evaluate differences among groups. *P*-values < 0.05 were considered to be statistically significant.

## Results and Discussion

### Effects of Baking on the Non-volatiles and the Color of Infusion

**FIGURE 1:**
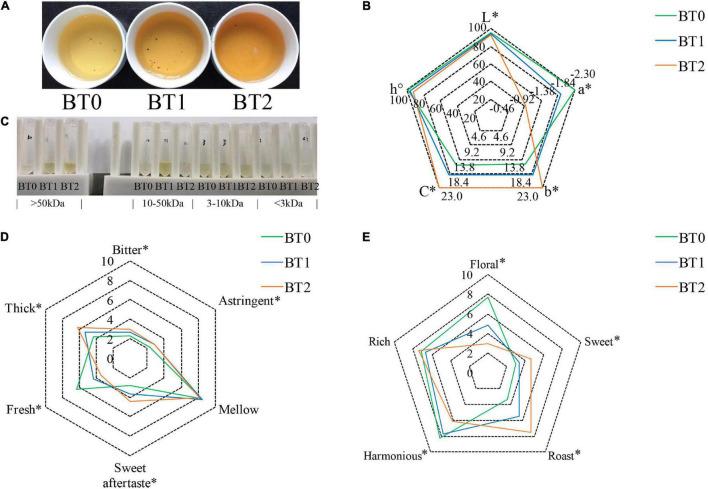
Sensory properties of jade (BT0), medium baked (BT1), and deep baked (BT2) *Tieguanyin* oolong teas. **(A)** Photo of tea infusions; **(B)** the color analysis determined by a spectrophotometer; **(C)** photo of tea infusion constituents separated using centrifugal filters with 3, 10, and 50 kDa molecular weight cut-off, accordingly; **(D)** taste characteristics and scores; and **(E)** aroma characteristics and scores. *P < 0.05 indicates significant difference.

The color of the infusion is an aspect to assess the sensory quality of tea. Sensory evaluation (Figure 1A) indicated that the baking process significantly darkened the color of tea infusion, turning it from honey yellow, golden yellow, to orange. Instrumental analysis of color (Figure 1B) showed that the a* value turned from −2.31 to −0.96, the b* value increased from 15.4 to 23.0, and the L* value decreased from 95.6 to 92.6 after twice baking. It indicated that the baking process brought more red and yellow tones but less luminosity to TOT infusion, which was in accordance with the results of the visual observation. Separating the tea infusion using centrifugal filters with 3, 10, and 50 kDa molecular weight cut-off (MWCO), accordingly, it was found that the color differences among the three tea infusions were mainly attributed to constituents that could not pass through the 10 kDa MWCO filter (Figure 1C). The orange/golden yellow pigments in BT2 had higher molecular weights than in BT1, suggesting that the baking process promoted the formation of colored macromolecules.

Previous studies revealed that flavonol glycosides and tea pigments (e.g., TFs, TRs, and TBs) contributed to the color of tea infusion (20). As flavonol glycosides are small molecules, it is speculated that TRs and TBs may cause the differences on the color of TOT infusions. TRs and TBs are heterogeneous water-soluble polymers of catechins. TRs, whose average molecular weight range from ∼700 to 40,000 Da, usually present red color. TBs, characterized by their high molecular weight and complex structure, usually present brown color and are negatively related to the brightness of tea infusions. BT2 contained higher TRs and TBs than BT0 and BT1, and BT1 contained higher TBs than BT0 (Table 1). Deducing from the fact that the content of TRs in BT1 was insignificantly different from that in BT0, but the redness was enhanced in the BT1 infusion (Figure 1B), there might be something else attributing to the redness of TOT infusions besides TRs. The content of TBs was increased while the brightness of tea infusion was decreased as the baking degree increased, demonstrating that TBs reduced the brightness of TOT infusions. The cause of the increase of TRs and TBs might be the accelerated oxidization and polymerization of catechins induced by the heat and aerobic environment during the baking process (21). A 7.4 and 17.4% loss of total monomeric catechins were detected in BT1 and BT2 (Table 2), respectively, supporting the hypothesis.

**TABLE 1:** The chemical compositions of jade (BT0), medium-baked (BT1), and deep-baked (BT2) *Tieguanyin* oolong teas.

Contents (mg/g)	BT0	BT1	BT2
Polyphenols	94.76 ± 4.52^a^	94.60 ± 6.14^a^	85.33 ± 0.84^b^
Free amino acids	28.69 ± 0.88^a^	20.49 ± 0.15^b^	12.70 ± 0.00^c^
Soluble proteins	28.99 ± 0.67^a^	28.36 ± 0.69^ab^	27.50 ± 0.66^b^
Soluble sugars	77.28 ± 0.77^a^	76.19 ± 0.77^a^	72.94 ± 1.92^b^
Soluble polysaccharides	15.95 ± 0.77^b^	16.46 ± 2.97^ab^	19.10 ± 1.08^a^
Flavones	8.53 ± 0.30^ab^	8.16 ± 0.23^b^	9.12 ± 0.68^a^
TFs	0.47 ± 0.11^ab^	0.37 ± 0.01^b^	0.41 ± 0.00^a^
TRs	12.36 ± 0.33^b^	12.40 ± 0.30^b^	13.01 ± 0.23^a^
TBs	10.07 ± 0.40^c^	11.04 ± 0.23^b^	12.31 ± 0.03^a^

*The same letter within each row indicates no significant difference (P > 0.05).*

**TABLE 2:** The contents of monomeric catechins, gallic acid, and caffeine in jade (BT0), medium-baked (BT1), and deep-baked (BT2) *Tieguanyin* oolong teas.

Contents (mg/g)	BT0	BT1	BT2
Gallic acid (GA)	1.03 ± 0.01^c^	1.22 ± 0.02^b^	1.36 ± 0.01^a^
Gallocatechin (GC)	12.33 ± 0.49^a^	8.25 ± 0.24^b^	7.58 ± 0.03^c^
Epigallocatechin (EGC)	22.06 ± 1.20^a^	21.82 ± 0.21^a^	18.65 ± 0.10^b^
Catechin (C)	1.59 ± 0.01^a^	1.38 ± 0.01^b^	1.17 ± 0.01^c^
Caffeine	16.73 ± 0.16^b^	17.05 ± 0.06^a^	16.54 ± 0.12^b^
Epigallocatechin gallate (EGCG)	38.01 ± 1.66^a^	37.45 ± 0.35^a^	34.69 ± 0.71^b^
Epicatechin (EC)	7.10 ± 0.10^a^	6.90 ± 0.04^b^	5.64 ± 0.01^c^
Gallocatechin gallate (GCG)	8.75 ± 0.82^a^	7.29 ± 0.24^b^	6.47 ± 0.28^c^
Epicatechin gallate (ECG)	7.64 ± 0.09^a^	7.25 ± 0.21^b^	6.18 ± 0.13^c^
Catechin gallate (CG)	0.62 ± 0.00^a^	0.49 ± 0.03^b^	0.35 ± 0.01^c^
Total monomeric catechins	98.09 ± 4.35^a^	90.83 ± 0.90^b^	80.74 ± 0.72^c^

*The same letter within each row indicates no significant difference (P > 0.05).*

### Effects of Baking on the Non-volatiles and Taste

Taste is the most crucial aspect of oolong tea’s sensory quality, based on GB/T 23776-2018. Sensory evaluation indicated that the baking process remarkably increased the thickness, sweet aftertaste, and bitterness, while reducing TOT infusion’s umami taste (Figure 1D).

Soluble polysaccharides were candidate contributors to the thickness of tea infusions. A 20% increase in the content of soluble polysaccharides was detected in BT2 (Table 1). It was reported that some polysaccharides increased the kokumi sensation (22), a taste impression combined of thickness, mouthfulness, and continuity, as they influenced the viscosity of fluids (23). Tea that tasted smooth and thick, such as ripe Pu’er tea and aged white tea, usually had abundant soluble polysaccharides. Heat treatment could promote the degradation of insoluble polysaccharides, thereby increasing the content of soluble polysaccharides in tea infusions. It was possible that the increase of soluble polysaccharides after baking also contributed to the thick taste. TRs might have an impact on the thickness of TOT infusions as well, because TRs were previously found to contribute to the mouth feel (thickness) (24).

**FIGURE 2:**
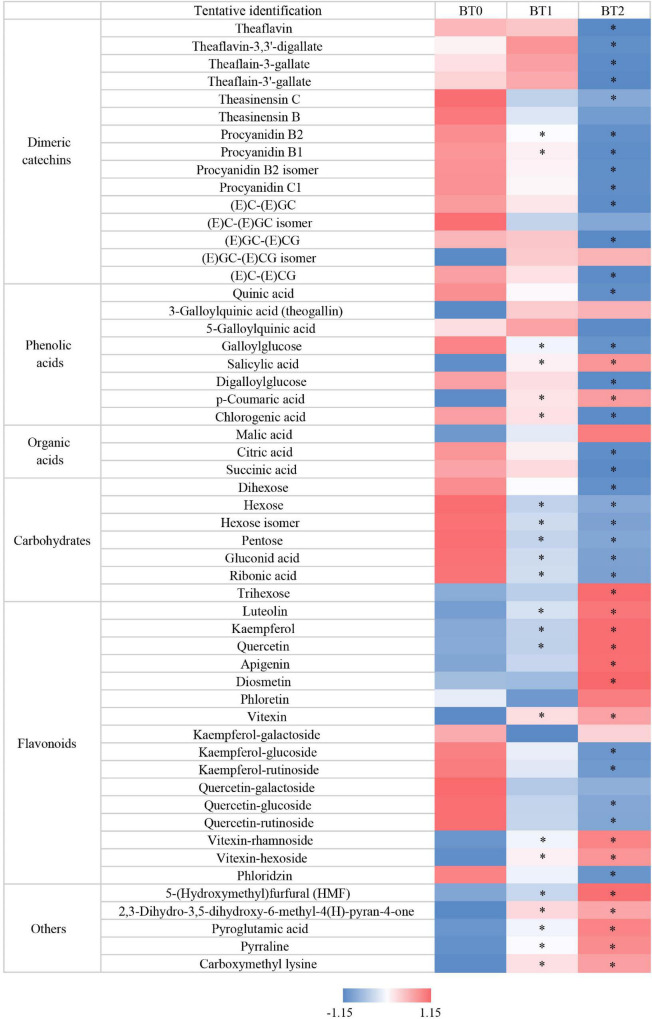
Heatmap of *Z*-score normalized relative abundances of non-volatiles identified by UPLC-QE-Orbitrap-MS. *P < 0.05 indicates significant difference.

Gallic acid, a degradation product of catechins, was associated with the sweet aftertaste of TOT infusions. Gallic acid was previously reported to improve the sweet aftertaste of tannase-treated autumn green tea (25). In this study, a 18 and 32% increase of gallic acid were detected in BT1 and BT2, respectively (Table 2), which was consistent with the gradual enhancement of sweet aftertaste after baking. Along with the increase of gallic acid, was the decrease of catechins. Among the eight catechins analyzed by HPLC, six were decreased after the first baking process, and eight were reduced after the second baking. Several dimeric catechins (e.g., theasinensins and procyanidins) were reduced after baking (Figure 2). In details, about 30% of TFs, 10% of theasinensins, and 30% of procyanidins were lost after twice baking. In a previous study, high-temperature processing (roasting) during tea production decreased monomeric catechins and increased gallic acid (26). Our results supported that the degradation of catechins were universal during the baking process of tea. Catechins and their derivatives are essential to the taste of oolong tea by enhancing bitterness and astringency (14). Monomeric catechins usually taste astringent and bitter, while dimeric catechins taste more astringent and less bitter than monomeric catechins (11). The bitterness and astringency were not reduced though catechins were decreased after baking (Figure 1D). On the contrary, both sensory attributes were enhanced, implying that other bitter and astringent compounds might be generated during the baking process.

It was assumed that the Maillard reaction was responsible to the bitterness of baked TOT infusions. The Maillard reaction is a chemical reaction between reducing sugars and amino acids upon the baking process, which produces a series of flavor compounds and modifies the sensory properties (e.g., bitterness) of baked foods (27, 28). Previous research demonstrated that the Maillard reaction impacted the quality of green tea and oolong tea (10, 29). In this study, 55% of total free amino acids was consumed after twice baking (Table 1). The contents of dihexose, hexoses, and pentose significantly decreased in baking. At the same time, the levels of 5-hydroxymethylfurfural (5-HMF) and 2,3-dihydro-3,5-dihydroxy-6-methyl-4(H)-pyran-4-one (DDMP), two typical intermediates of Maillard reaction with a bitter taste (30), in BT2 was 7.7-fold and 2.6-fold of that in BT0. These data proved the presence of the Maillard reaction during the baking process of TOT and its role in the chemical and taste changes of baked TOTs.

It was aware that the occurrence of the Maillard reaction was accompanied by the consumption of free amino acids. A total of 17 free amino acids were detected, including 14 proteinogenic amino acids and 3 non-protein amino acids (i.e., theanine, β-aminoisobutyric acid, and γ-aminobutyric acid) (Table 3). Theanine and glutamic acid, major umami compounds in tea (31), were the two most abundant free amino acids. Together, the two accounted for about 60% of total free amino acids in TOT infusions. However, merely 24.3% of theanine and 32.7% of glutamic acid remained after baking twice, which led to the reduced umami taste of baked TOT infusions (Figure 1D). Our previous study suggested that the free amino acid content was positively correlated to the taste quality of TOTs (1). The baking-induced decrease of free amino acids might hamper the taste of TOTs, but benefit the formation of typical roast aroma *via* the Maillard reaction.

**TABLE 3:** The contents of free amino acid components in jade (BT0), medium-baked (BT1), and deep-baked (BT2) *Tieguanyin* oolong teas.

Content (μg/g)	BT0	BT1	BT2
Aspartate	1,047.4 ± 54.8^a^	893.8 ± 255.3^a^	576.8 ± 61.0^b^
Threonine	576.1 ± 34.5^a^	456.5 ± 64.6^b^	232.2 ± 17.2^c^
Serine	1,447.7 ± 29.7^a^	1,107.9 ± 121.7^b^	508.8 ± 55.5^c^
Asparagine	436.5 ± 32.2^a^	385.9 ± 167.5^a^	21.0 ± 3.2^b^
Glutamic acid	3,266.5 ± 211.5^a^	2,044.9 ± 226.0^b^	1,066.7 ± 33.7^c^
Theanine	18,030.3 ± 2,679.5^a^	11,112.8 ± 1,612.5^b^	4,382.8 ± 214.4^c^
Glycine	94.7 ± 4.5^a^	80.5 ± 4.7^b^	51.9 ± 1.2^c^
Alanine	1,258.8 ± 45.8^a^	1,064.3 ± 85.3^b^	564.4 ± 7.9^c^
Valine	628.1 ± 35.0^a^	487.6 ± 60.6^b^	270.9 ± 6.2^c^
Cystine	96.5 ± 6.1^a^	67.2 ± 7.4^b^	21.5 ± 1.5^c^
Isoleucine	113.6 ± 14.1^a^	84.0 ± 22.3^b^	36.4 ± 3.8^c^
Leucine	146.2 ± 17.2^a^	107.8 ± 22.9^a^	52.3 ± 0.3^b^
Tyrosine	305.0 ± 4.6^a^	243.6 ± 39.2^b^	124.7 ± 17.3^c^
Phenylalanine	158.5 ± 17.3^a^	142.7 ± 11.5^a^	112.3 ± 6.0^b^
β-Aminoisobutyric acid	257.0 ± 68.9^a^	191.7 ± 32.2^a^	77.7 ± 6.2^b^
γ-Aminobutyric acid	178.8 ± 10.5^a^	112.4 ± 20.4^a^	49.3 ± 3.6^b^
Lysine	223.0 ± 19.4^a^	198.3 ± 19.9^a^	90.7 ± 15.7^b^

*The same letter within each row indicates no significant difference (P > 0.05).*

### Effects of Baking on the Volatiles and Aroma

Aroma is the second important aspect of oolong tea’s sensory quality, based on GB/T 23776-2018. Sensory evaluation indicated that the baking process transformed the aroma from floral to sweet and roast (Figure 1E).

The changes of volatiles after baking were the basis of the changes in the aroma. Table 4 indicated that the baking process significantly increased the number of volatiles. A total of 74, 85, and 102 volatiles were identified in BT0, BT1, and BT2, respectively. Among them, 61 volatiles were detected in all three samples. BT2 contained more unique volatiles than the other two. It indicated that the baking process promoted the formation of new volatiles, many of which belonged to heterocycles.

**TABLE 4:** Volatile compounds in jade (BT0), medium-baked (BT1), and deep-baked (BT2) *Tieguanyin* oolong teas determined by GC-MS.

Retention time	CAS number	Molecular formula	Molecular weight	Name	Relative abundance%			Aroma properties
					BT0	BT1	BT2	
1.642	75-18-3	C_2_H_6_S	62	Dimethyl sulfide	0.342 ± 0.056^a^	0.292 ± 0.051^a^	<LOQ ^b^	Sulfury
1.732	79-20-9	C_3_H_6_O_2_	74	Methyl acetate	<LOQ ^b^	<LOQ ^b^	0.526 ± 0.170^a^	Sweet fruity
1.741	1191-16-8	C_7_H_12_O_2_	128	Prenyl acetate	<LOQ ^b^	0.440 ± 0.006^a^	<LOQ ^b^	Sweet banana, fruity
1.800	78-84-2	C_4_H_8_O	72	Isobutyraldehyde	<LOQ ^b^	0.069 ± 0.008^a^	0.073 ± 0.009^a^	Malty, aldehydic
2.023	111-30-8	C_5_H_8_O_2_	100	Glutaraldehyde	0.060 ± 0.009^b^	0.089 ± 0.010^a^	0.105 ± 0.007^a^	Pungent
2.077	534-22-5	C_5_H_6_O	82	2-Methylfuran	0.080 ± 0.012^b^	0.083 ± 0.010^b^	0.147 ± 0.027^a^	Ethereal, acetone, chocolate
2.329	NIST#: 194652	C_7_H_10_O_2_	126	3-Methyl-4-propenyl-oxetan-2-one	0.052 ± 0.004^a^	0.050 ± 0.007^a^	0.054 ± 0.003^a^	/
2.376	926-54-5	C_6_H_10_	82	Trans-2-methyl-1,3-pentadiene	0.089 ± 0.006^a^	0.068 ± 0.002^b^	<LOQ ^c^	/
2.445	96-38-8	C_6_H_8_	80	5-Methyl-1,3-cyclopentadiene	<LOQ ^b^	<LOQ ^b^	0.032 ± 0.005^a^	/
2.479	590-86-3	C_5_H_10_O	86	3-Methylbutanal	0.074 ± 0.021^a^	0.044 ± 0.005^b^	0.055 ± 0.007^ab^	Fruity, pungent, nutty, cocoa
2.582	96-17-3	C_5_H_10_O	86	2-Methylbutanal	0.179 ± 0.031^a^	0.135 ± 0.018^a^	0.225 ± 0.025^a^	Musty, cocoa, coffee, nutty
2.816	96-41-3	C_5_H_10_O	86	Cyclopentanol	<LOQ ^b^	<LOQ ^b^	0.075 ± 0.024^a^	Musty, aromatic
2.888	1629-58-9	C_5_H_8_O	84	1-Penten-3-one	0.238 ± 0.027^a^	0.252 ± 0.047^a^	0.167 ± 0.025^b^	Pungent, peppery, garlic
3.073	589-91-3	C_7_H_14_O	114	4-Methylcyclohexanol	0.576 ± 0.075^a^	0.487 ± 0.021^a^	0.375 ± 0.027^b^	Aromatic
3.182	50521-50-1	C_6_H_10_O_2_	114	1,4-Butanediol, 2,3-bis(methylene)-	<LOQ ^b^	<LOQ ^b^	0.041 ± 0.002^a^	/
3.415	30316-00-8	C_7_H_11_N	109	2-Methyl-5-hexenenitrile	0.038 ± 0.007^ab^	0.059 ± 0.013^a^	0.035 ± 0.007^b^	/
3.480	1943-79-9	C_8_H_9_NO_2_	151	Methylcarbamic acid phenyl ester	<LOQ ^b^	<LOQ ^b^	0.071 ± 0.011^a^	/
3.633	625-28-5	C_5_H_9_N	83	Isovaleronitrile	0.035 ± 0.008^c^	0.060 ± 0.007^b^	0.130 ± 0.009^a^	/
3.761	96-54-8	C_5_H_7_N	81	1-Methyl-pyrrole	<LOQ ^c^	0.026 ± 0.001^b^	0.046 ± 0.004^a^	Smoky, woody
3.883	497-03-0	C_5_H_8_O	84	(E)-2-Methyl-2-butenal	<LOQ ^b^	0.054 ± 0.010^a^	0.060 ± 0.009^a^	Strong green
4.022	21856-89-3	C_6_H_12_O_2_	116	6-Hydroxyhexan-2-one	<LOQ ^b^	<LOQ ^b^	0.077 ± 0.013^a^	/
4.075	55230-25-6	C_6_H_10_O	98	2-Methyl-5,6-dihydro-2H-pyran	<LOQ ^c^	0.069 ± 0.006^b^	0.108 ± 0.015^a^	/
4.168	89182-08-1	C_5_H_8_O	84	Cyclobut-1-enylmethanol	0.368 ± 0.057^a^	0.359 ± 0.012^a^	0.413 ± 0.053^a^	/
4.420	108-88-3	C_7_H_8_	92	Toluene	0.154 ± 0.030^c^	0.278 ± 0.035^b^	0.695 ± 0.119^a^	Sweet
4.837	4054-38-0	C_7_H_10_	94	1,3-Cycloheptadiene	0.073 ± 0.003^a^	0.063 ± 0.002^b^	0.060 ± 0.012^ab^	/
5.302	141-79-7	C_6_H_10_O	98	Mesityl oxide	<LOQ ^b^	0.132 ± 0.029^a^	<LOQ ^b^	Honeylike
5.381	66-25-1	C_6_H_12_O	100	Hexanal	2.563 ± 0.419^a^	2.111 ± 0.326^a^	1.502 ± 0.204^b^	Fresh green
5.689	617-92-5	C_6_H_9_N	95	1-Ethyl pyrrole	0.573 ± 0.103^b^	1.529 ± 0.197^a^	1.707 ± 0.188^a^	Burnt
6.433	98-01-1	C_5_H_4_O_2_	96	Furfural	0.434 ± 0.068^c^	2.102 ± 0.280^b^	5.295 ± 0.368^a^	Sweet, woody, baked bread
7.330	6728-26-3	C_6_H_10_O	98	(E)-2-Hexenal	0.402 ± 0.059^a^	0.400 ± 0.061^a^	0.253 ± 0.010^b^	Green banana, fatty
7.962	106-42-3	C_8_H_10_	106	p-Xylene	0.084 ± 0.018^c^	0.191 ± 0.006^b^	1.162 ± 0.120^a^	Aromatic
8.970	110-43-0	C_7_H_14_O	114	2-Heptanone	0.271 ± 0.027^b^	0.332 ± 0.052^ab^	0.379 ± 0.054^a^	Fruity
9.467	6728-31-0	C_7_H_12_O	112	(Z)-4-Heptenal	0.181 ± 0.032^a^	0.191 ± 0.034^a^	0.173 ± 0.015^a^	Green, creamy
9.607	111-71-7	C_7_H_14_O	114	Heptanal	0.404 ± 0.019^a^	0.282 ± 0.035^b^	0.255 ± 0.031^b^	Green, fatty
9.706	2199-41-9	C_7_H_11_N	109	2,3,5-Trimethyl-1H-pyrrole	<LOQ ^c^	0.201 ± 0.046^b^	0.286 ± 0.023^a^	/
9.895	1192-62-7	C_6_H_6_O_2_	110	2-Acetyl furan	<LOQ ^c^	0.124 ± 0.016^b^	0.262 ± 0.013^a^	Caramel, sweet
10.086	1558-17-4	C_6_H_8_N_2_	108	4,6-dimethyl-pyrimidine	<LOQ ^c^	0.199 ± 0.032^b^	0.301 ± 0.025^a^	/
10.309	56342-53-1	C_6_H_8_N_2_	108	1-Methyl-3-vinyl-1H-pyrazole	<LOQ ^b^	0.140 ± 0.018^a^	0.179 ± 0.024^a^	/
10.511	930-87-0	C_7_H_11_N	109	1,2,5-Trimethylpyrrole	<LOQ ^c^	0.132 ± 0.023^b^	0.165 ± 0.002^a^	/
10.834	106-70-7	C_7_H_14_O_2_	130	Methyl hexanoate	0.111 ± 0.016^a^	0.111 ± 0.006^a^	0.126 ± 0.008^a^	Fruity
11.197	2396-78-3	C_7_H_12_O_2_	128	Methyl 3-hexenoate	<LOQ ^c^	0.074 ± 0.013^b^	0.165 ± 0.006^a^	Earthy, sweet, slightly fruity
11.860	589-33-3	C_8_H_13_N	123	1-Butylpyrrole	<LOQ ^b^	<LOQ ^b^	0.034 ± 0.010^a^	/
12.746	100-52-7	C_7_H_6_O	106	Benzaldehyde	2.992 ± 0.322^b^	3.651 ± 0.060^a^	3.844 ± 0.165^a^	Bitter almond, cherry
13.614	611-13-2	C_6_H_6_O_3_	126	Methyl 2-furoate	<LOQ ^b^	<LOQ ^b^	0.272 ± 0.038^a^	Caramel
14.389	110-93-0	C_8_H_14_O	126	6-Methyl-5-hepten-2-one	2.451 ± 0.453^a^	2.131 ± 0.369^a^	2.006 ± 0.054^a^	Green, lemongrass, citrus
14.692	3777-69-3	C_9_H_14_O	138	2-Pentyl-furan	0.747 ± 0.310^a^	0.627 ± 0.061^a^	<LOQ ^b^	Green, beany
14.697	80255-20-5	C_9_H_13_NO_2_	167	1-(2-Nitro-2-propenyl)-cyclohexene	<LOQ ^b^	<LOQ ^b^	0.937 ± 0.064^a^	/
15.071	4313-03-5	C_7_H_10_O	110	(E,E)-2,4-Heptadienal	6.334 ± 0.175^c^	9.387 ± 0.823^b^	11.103 ± 0.022^a^	Fatty, green
15.573	124-13-0	C_8_H_16_O	128	Octanal	0.494 ± 0.081^a^	0.446 ± 0.045^a^	<LOQ ^b^	Citrus
15.581	513-23-5	C_10_H_18_O	154	Isothujol	<LOQ ^b^	<LOQ ^b^	0.573 ± 0.090^a^	/
16.770	527-84-4	C_10_H_14_	134	o-Cymene	0.666 ± 0.068^ab^	0.543 ± 0.087^b^	0.768 ± 0.071^a^	Citrus
17.072	5989-27-5	C_10_H_16_	136	D-Limonene	3.924 ± 0.331^a^	3.333 ± 0.396^ab^	2.907 ± 0.500^b^	Lemon, citrus
17.386	2408-37-9	C_9_H_16_O	140	2,2,6-Trimethyl-cyclohexanone	0.169 ± 0.020^ab^	0.141 ± 0.023^b^	0.185 ± 0.008^a^	Pungent, citrus
17.759	3338-55-4	C_10_H_16_	136	(Z)-β-Ocimene	<LOQ ^b^	<LOQ ^b^	0.290 ± 0.019^a^	Warm floral, sweet
17.963	122-78-1	C_8_H_8_O	120	Benzeneacetaldehyde	5.576 ± 0.401^a^	3.804 ± 0.345^b^	1.700 ± 0.068^c^	Hyacinth, sweet floral
18.216	1877-77-6	C_7_H_9_NO	123	3-Amino-benzenemethanol	<LOQ ^c^	2.851 ± 0.056^b^	4.065 ± 0.121^a^	/
18.248	264628-15-1	C_9_H_12_	120	5-Methylenecycloocta-1,3-diene	1.549 ± 0.034^a^	<LOQ ^b^	< *LOQ*^b^	/
18.418	5794-03-6	C_10_H_16_	136	(+)-Camphene	<LOQ ^b^	0.788 ± 0.124^a^	0.796 ± 0.029^a^	Camphor, fresh herbal
18.423	7785-70-8	C_10_H_16_	136	D-(+)-α-Pinene	1.095 ± 0.043^a^	<LOQ ^b^	<LOQ ^b^	Harsh, terpene, aromatic
18.914	14296-81-2	C_9_H_12_	120	Cyclohexane, 1,2,4-tris(methylene)-	0.400 ± 0.031^a^	0.462 ± 0.183^a^	0.392 ± 0.032^a^	/
19.162	2548-87-0	C_8_H_14_O	126	(E)-2-Octenal	0.480 ± 0.061^b^	0.644 ± 0.100^b^	0.740 ± 0.017^a^	Fatty, fresh cucumber
19.363	41898-89-9	C_9_H_12_	120	2,4-Dimethyl-2,3-heptadien-5-yne	<LOQ ^c^	0.299 ± 0.044^b^	0.450 ± 0.051^a^	/
19.932	30086-02-3	C_8_H_12_O	124	(E,E)-3,5-Octadien-2-one	1.783 ± 0.108^c^	2.188 ± 0.078^b^	2.418 ± 0.136^a^	Fruity, green, grassy
20.280	NIST#: 129149	C_8_H_12_N_2_	136	Imidazole, 4-methyl-5-[2-methyl-2-propenyl]-	<LOQ ^b^	<LOQ ^b^	0.385 ± 0.032^a^	/
20.878	514-95-4	C_10_H_16_	136	1,5,5-Trimethyl-6-methylene-cyclohexene	<LOQ ^b^	<LOQ ^b^	0.389 ± 0.030^a^	/
20.879	NIST#: 274055	C_14_H_20_O_3_	236	Acetic acid, 2-(7-methylenebicyclo[3.3.1] oct-2-enyloxy)ethyl ester	0.240 ± 0.046^a^	0.297 ± 0.053^a^	<LOQ ^b^	/
21.049	5989-33-3	C_10_H_18_O_2_	170	(Z)-Linalool oxide (furanoid)	0.141 ± 0.028^c^	0.205 ± 0.036^b^	0.272 ± 0.014^a^	Earthy, floral sweet, woody
21.180	1195-32-0	C_10_H_12_	132	1-Methyl-4-(1-methylethenyl)-benzene	<LOQ ^b^	<LOQ ^b^	0.220 ± 0.030^a^	Phenolic, spicy, guaiacol
21.623	56846-98-1	C_19_H_30_O_2_	290	13,16-Octadecadiynoic acid, methyl ester	<LOQ ^b^	<LOQ ^b^	0.414 ± 0.048^a^	/
21.631	28638-29-1	C_9_H_18_O	142	2,3,4-Trimethyl-5-hexen-3-ol	0.247 ± 0.033^b^	0.379 ± 0.055^a^	<LOQ ^c^	/
22.079	78-70-6	C_10_H_18_O	154	Linalool	1.535 ± 0.216^a^	1.130 ± 0.141^b^	0.762 ± 0.037^c^	Floral, sweet, citrus
22.267	29957-43-5	C_10_H_16_O	152	Dehydrolinalool	3.362 ± 0.294^b^	5.533 ± 0.532^a^	2.762 ± 0.171^c^	/
22.420	124-19-6	C_9_H_18_O	142	Non-anal	0.728 ± 0.004^a^	0.623 ± 0.116^ab^	0.526 ± 0.041^b^	Rose, orange, waxy
22.604	50868-73-0	C_8_H_11_NO	137	2-Methoxy-6-methylaniline	<LOQ ^c^	0.740 ± 0.098^b^	1.182 ± 0.181^a^	/
23.028	19945-61-0	C_11_H_18_	150	(E)-4, 8-Dimethyl-1,3,7-non-atriene	1.735 ± 0.097^a^	1.132 ± 0.188^b^	0.796 ± 0.044^c^	/
24.016	1079-01-2	C_12_H_18_O_2_	194	Myrtenyl acetate	0.241 ± 0.011^a^	<LOQ ^b^	<LOQ ^b^	Herbal, fresh
24.417	140-29-4	C_8_H_7_N	117	Phenyl acetonitrile	4.060 ± 0.188^c^	5.946 ± 0.449^b^	7.726 ± 0.456^a^	Aromatic
24.867	3682-17-5	C_9_H_9_NO_3_	179	α-(Hydroxyimino)-benzenepropanoic acid	0.726 ± 0.115^b^	0.592 ± 0.012^b^	1.217 ± 0.109^a^	/
25.993	73476-31-0	C_8_H_11_NO_2_	153	Methyl 1,5-dimethyl-2-pyrrolecarboxylate	<LOQ ^b^	<LOQ ^b^	0.218 ± 0.011^a^	/
26.377	91253-94-0	C_11_H_18_O	166	2-Naphthol, 1,2,3,4,4a,5,6,7-octahydro-4a-methyl-	<LOQ ^b^	<LOQ ^b^	0.253 ± 0.003^a^	/
27.364	22767-95-9	C_12_H_16_O_2_	192	Benzenepropanoic acid 1-methylethyl ester	<LOQ ^b^	<LOQ ^b^	0.350 ± 0.044^a^	/
27.492	1438-94-4	C_9_H_9_NO	147	1-Furfuryl pyrrole	<LOQ ^b^	<LOQ ^b^	1.072 ± 0.089^a^	Plastic, waxy, coffee
28.439	119-36-8	C_8_H_8_O_3_	152	Methyl salicylate	1.322 ± 0.074^b^	1.395 ± 0.292^b^	2.418 ± 0.136^a^	Wintergreen mint
28.990	99172-18-6	C_10_H_14_O	150	2-Ethylidene-6-methyl-3,5-heptadienal	0.408 ± 0.085^a^	0.367 ± 0.052^a^	0.413 ± 0.045^a^	/
30.424	432-25-7	C_10_H_16_O	152	β-Cyclocitral	0.670 ± 0.038^a^	0.583 ± 0.044^b^	0.625 ± 0.021^ab^	Tropical, saffron, herbal
31.709	35154-45-1	C_11_H_20_O_2_	184	cis-3-Hexenyl isovalerate	0.399 ± 0.052^a^	0.199 ± 0.029^b^	0.228 ± 0.018^b^	Fresh green apple
31.905	4677-90-1	C_14_H_20_O	204	Mayurone	<LOQ ^b^	<LOQ ^b^	0.066 ± 0.009^a^	/
32.111	10032-15-2	C_11_H_22_O_2_	186	Butanoic acid, 2- methyl-, hexyl ester	0.187 ± 0.016^a^	0.077 ± 0.024^b^	0.114 ± 0.018^b^	Green, spicy
32.931	472-66-2	C_11_H_18_O	166	β-Homocyclocitral	0.077 ± 0.009^a^	<LOQ ^b^	<LOQ ^b^	Cooling woody, camphor
33.134	6290-37-5	C_14_H_20_O_2_	220	Phenethyl hexanoate	0.199 ± 0.019^c^	0.279 ± 0.049^b^	0.416 ± 0.009^a^	Sweet, honey, floral
34.462	NIST#: 196695	C_13_H_20_O	192	1H-2-Indenone,2,4,5,6,7,7a-hexahydro-3-(1-methylethyl)-7a-methyl	<LOQ ^b^	<LOQ ^b^	0.406 ± 0.041^a^	/
35.280	120-72-9	C_8_H_7_N	117	Indole	14.935 ± 1.281^a^	11.255 ± 0.990^b^	6.260 ± 1.066^c^	Floral
35.739	6125-24-2	C_8_H_9_NO_2_	151	2-Nitroethyl-benzene	3.588 ± 0.199^a^	3.483 ± 0.130^a^	<LOQ ^b^	Floral, spicy
35.761	700-88-9	C_11_H_14_	146	Cyclopentylbenzene	<LOQ ^b^	<LOQ ^b^	2.508 ± 0.104^a^	/
36.624	35845-67-1	C_13_H_18_O	190	3,6-Nonadien-5-one, 2,2,8,8-tetramethyl-	<LOQ ^b^	<LOQ ^b^	0.510 ± 0.030^a^	/
38.889	30364-38-6	C_13_H_16_	172	Dehydro-ar-ionene	0.092 ± 0.003^c^	0.176 ± 0.024^b^	0.567 ± 0.041^a^	Licorice
39.037	475-03-6	C_13_H_18_	174	α-Ionene	0.066 ± 0.001^b^	0.067 ± 0.004^b^	0.129 ± 0.007^a^	Grassy
40.805	63435-25-6	C_13_H_18_	174	Benzene, 2-(2-butenyl)-1,3,5-trimethyl-	<LOQ ^b^	<LOQ ^b^	0.096 ± 0.003^a^	/
40.963	31501-11-8	C_12_H_22_O_2_	198	(Z)-3-Hexen-1-yl hexanoate	1.970 ± 0.077^a^	1.461 ± 0.185^b^	1.215 ± 0.056^b^	Fruity, green
41.308	6378-65-0	C_12_H_24_O_2_	200	Hexyl hexanoate	0.837 ± 0.035^a^	0.681 ± 0.046^b^	0.447 ± 0.032^c^	Fresh cut grass
41.470	488-10-8	C_11_H_16_O	164	Jasmone	0.805 ± 0.047^a^	0.917 ± 0.093^a^	0.543 ± 0.088^b^	Floral, jasmine, woody, herbal
42.615	87-44-5	C_15_H_24_	204	Caryophyllene	0.100 ± 0.007^a^	0.091 ± 0.014^a^	<LOQ ^b^	Woody, spicy, clove
42.968	6901-97-9	C_13_H_20_O	192	α-Ionone	0.167 ± 0.019^b^	0.195 ± 0.013^ab^	0.214 ± 0.016^a^	Floral
43.225	NIST#: 187519	C_13_H_18_O	190	4-(2,4,4-Trimethyl-cyclohexa-1,5-dienyl)-but-3-en-2-one	0.113 ± 0.010^b^	0.306 ± 0.038^a^	0.339 ± 0.009^a^	/
43.968	103-52-6	C_12_H_16_O_2_	192	β-Phenylethyl butyrate	0.352 ± 0.038^a^	0.362 ± 0.047^a^	0.338 ± 0.047^a^	Sweet floral
44.550	3879-26-3	C_13_H_22_O	194	Neryl acetone	0.276 ± 0.031^a^	0.242 ± 0.042^a^	0.302 ± 0.029^a^	Fatty, metallic
44.808	18794-84-8	C_15_H_24_	204	(E)-β-Famesene	0.774 ± 0.138^a^	0.674 ± 0.082^a^	0.781 ± 0.132^a^	Woody
45.148	4602-84-0	C_15_H_26_O	222	Farnesol	<LOQ ^b^	<LOQ ^b^	0.135 ± 0.020^a^	Mild fresh sweet
45.938	14901-07-6	C_13_H_20_O	192	β-Ionone	0.719 ± 0.072^a^	0.818 ± 0.099^a^	0.519 ± 0.020^b^	Floral, woody
46.093	81968-62-9	C_15_H_24_O	220	(1R,7S,E)-7-Isopropyl-4,10-dimethylenecyclodec-5-enol	0.731 ± 0.111^a^	<LOQ ^b^	0.667 ± 0.025^a^	/
46.310	7460-74-4	C_13_H_18_O_2_	206	2-Phenylethyl valerate	0.540 ± 0.062^a^	0.427 ± 0.077^ab^	0.392 ± 0.036^b^	Fruity, rose
46.784	13474-59-4	C_15_H_24_	204	trans-α-Bergamotene	0.553 ± 0.003^a^	0.402 ± 0.144^ab^	0.316 ± 0.032^b^	Woody, warm, tea
47.000	6892-80-4	C_15_H_26_O	222	Widdrol	<LOQ ^b^	<LOQ ^b^	0.300 ± 0.060^a^	/
47.173	25524-95-2	C_10_H_16_O_2_	168	(Z)-7-Decen-5-olide	0.664 ± 0.082^a^	<LOQ ^b^	< *LOQ*^b^	Creamy, jasmine
47.443	502-61-4	C_15_H_24_	204	α-Farnesene	3.635 ± 0.287^a^	2.630 ± 0.222^b^	2.112 ± 0.097^c^	Herbal, citrus
50.173	40716-66-3	C_15_H_26_O	222	(E)-Nerolidol	16.85 ± 1.533^a^	13.879 ± 1.656^a^	7.859 ± 0.679^b^	Floral

*The same letter within each row indicates no significant difference (P > 0.05). LOQ is short for the limit of quantification.*

Floral volatiles were the main volatiles detected in BT0. Although the number of floral volatiles did not vary considerably among the three teas, their relative abundances did. The relative abundances of floral volatiles accounted for 46.6% of total volatiles in BT0. (E)-Nerolidol, indole, and benzeneacetaldehyde were the top three abundant floral volatiles in BT0, making up 37.4% of total volatiles. The baking process decreased the contents of floral volatiles. The loss of floral volatiles occurred during the first and second baking stages, but was more severe during the second stage. The relative abundance of floral volatiles dropped to 19.7% in BT2. Previous study indicated that high temperature hampered the floral volatiles in TOT, i.e., β-ionone, jasmine, and nerolidol (32). Our results were partially in accordance with it. The differences might be caused by the differences in the raw material and the conditions of heat treatment. In addition, multiple green volatiles [e.g., cis-3-hexenyl isovalerate, heptanal, hexyl hexanoate, (Z)-3-hexen-1-yl hexanoate, hexanal, and 2-pentyl-furan], citrus volatiles (e.g., octanal and D-limonene), and herbal volatiles (e.g., α-farnesene and myrtenyl acetate) were reduced or disappeared after baking. The sum of the relative abundances of green/citrus/herbal volatiles decreased from 16.4 to 8.7%, leading to a less refreshing aroma in BT2.

Meanwhile, the types and relative abundances of sweet, caramel, and roast volatiles were significantly increased. Six, 12, and 14 sweet, caramel, and roast volatiles were identified in BT0, BT1, and BT2, accounting for 1.6, 5.3, and 11.0% of total volatiles, respectively. The relative abundances of toluene, phenethyl hexanoate, furfural, and 1-ethyl pyrrole gradually increased with baking. The relative abundances of these volatiles in BT2 were at least twice as much as that in BT0, respectively. Notably, the relative abundance of furfural, a volatile with a sweet and baked bready aroma and a typical intermediate of the Maillard reaction, was 11-fold higher in BT2 than that in BT0. Methyl 3-hexanoate, 2-acetyl furan, and 1-methyl-pyrrole, which existed in BT1 and BT2 but not in BT0, were enriched in BT2. Methyl acetate, farnesol, methyl 2-furoate, and 1-furfuryl pyrrole were merely detected in BT2. Earlier study revealed that 1-ethyl pyrrole was positively correlated with the grade of deep-baked TOT, while methyl acetate was initially decreased but then increased with the declining grade of deep-baked TOT (33). In this study, the two volatiles were accumulated in BT2, implying that the baking process was important for the formation of the aroma property of deep-baked TOT. It was noticed that many volatiles generated or accumulated during baking were pyrroles, furans, and their derivatives, which were Maillard reaction products, indicating that the baking process mainly reshaped the aroma *via* the Maillard reaction.

In addition to volatiles with known aroma characteristics, 27 volatiles detected in BT2 but not in BT0 were with unknown aroma characteristics. The contents of these compounds accounted for 14.4% of total volatiles. Most of them were complex structures, containing at least one cyclic ring. Little information on the aroma properties of these compounds could be found. It is necessary to use gas chromatography-olfactometry to investigate the aroma properties of these compounds and assess whether they contribute to the aroma of tea in further studies.

### Effects of Baking on the Chemical Composition and Bioactivity

**FIGURE 3:**
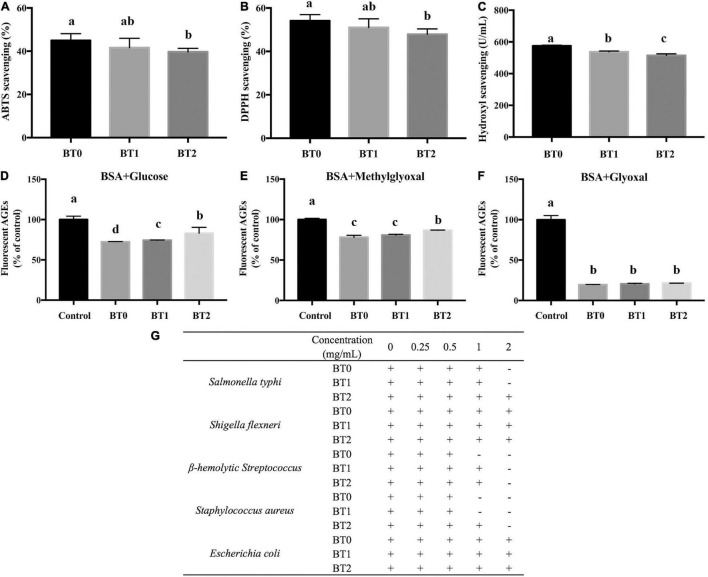
Bioactivities of jade (BT0), medium baked (BT1), and deep baked (BT2) *Tieguanyin* oolong teas. **(A)** ABTS scavenging activity; **(B)** DPPH scavenging activity; **(C)** hydroxyl scavenging activity; relative fluorescent AGEs formation in the bovine serum albumin (BSA) + glucose system **(D)**, BSA + methylglyoxal system **(E)**, and BSA + glyoxal system **(F)**; **(G)** minimum inhibitory concentrations of the three tea extracts against five bacteria. “+” means visible growth of the bacteria, while “–” means no visible bacterial growth. The same letter within each column indicates no significant difference (*P* > 0.05).

Chemical analysis revealed that the baking process dramatically altered the compositions and contents of catechins and their derivatives, flavones, free amino acids, and soluble sugars, many of which were bioactive compounds (Tables 1, 2, 3 and Figure 2). Hence, it was wondered whether the baking process also altered the bioactivity of TOTs. Antioxidant property is a featuring bioactivity of oolong tea. Free radicals are highly oxidizing and may damage macromolecules in the living system *via* inducing oxidative stress. Scavenging free radicals is an important strategy for antioxidants to exert their functions. Thus, free radical scavenging assays are often applied to evaluate the antioxidant activity. Recently, anti-AGEs formation assays are gaining attention as another way to evaluate antioxidant activity. Reactive oxygen species and free radicals participate in the formation of AGEs, and AGEs can induce oxidative stress *via* binding to their cell surface receptor (34). Many antioxidants possess inhibitory effects on the formation of AGEs (35). In this study, the above two assays were conducted to assess the antioxidant activity of three TOTs (Figures 3A–F). Antibacterial property is another featuring bioactivity of oolong tea. The MICs against five bacteria were used to determine the antibacterial activity (Figure 3G).

The effects of baking on the free radical scavenging activity are disadvantageous. The ABTS, DPPH, and hydroxyl radical scavenging activities of BT2 were significantly reduced compared with BT0 (Figures 3A–C). Earlier researches suggested that the ethyl acetate fraction of TOT, which had higher contents of phenolics, flavonoids, procyanidins, sugars, and catechin monomers, exhibited stronger antioxidant capacity than the n-butanol fraction and water fraction of TOT (36). Su et al. demonstrated that monomeric catechins played critical roles in anti-oxidation (37). It was reported that the trans-catechins possessed higher free radical scavenging activity than the corresponding cis-epicatechins, and the gallated catechins possessed higher antioxidant activity than the corresponding non-gallated catechins (38). Lee et al. tested the ABTS and DPPH scavenging activities of seven monomeric catechins. Compared with non-gallated catechins, gallated catechins did exhibit stronger ABTS and DPPH scavenging activities (39). Su et al. demonstrated that two major catechins, i.e., epigallocatechin gallate (EGCG) and epigallocatechin (EGC), contributed significantly to oolong tea’s DPPH and superoxide radical scavenging activities. Guo et al. investigated the hydroxyl radical scavenging activity of four epi-form catechins. The results indicated that their ability to scavenge hydroxyl radicals decreased in the order of epicatechin gallate (ECG) > epicatechin (EC) > EGCG > EGC (40). In our research, the changing trends of catechins contents, particularly the changing trends of epi-form catechins contents, were consistent with the changing trends of free radical scavenging activity during baking. It suggested that the decrease of catechins was an important cause of the baking-mediated decreased free radical scavenging activity of TOT.

The effects of baking on inhibiting the formation of AGEs are inconsistent in different reaction systems. Dietary AGEs are formed in foods during the thermal process, and they are a main source of AGEs in the body (41). The formation of AGEs begins between a carbonyl group of reducing sugar (e.g., glucose) and a free amino group, which generates an unstable Schiff base. It spontaneously cyclizes and undergoes the Amadori rearrangement, producing intermediates with highly reactive carbonyl groups, such as glyoxal and methylglyoxal (42). These products further react with free amino groups to form stable AGEs. Many AGEs are highly oxidant and pro-inflammatory, usually regarded as harmful to health (43). In this study, the baking process weakened the activity of TOTs in blocking the formation of AGEs in the BSA + glucose reaction system and the BSA + methylglyoxal reaction system, while hardly affecting that in the BSA + glyoxal reaction system (Figures 3D–3F). Earlier research indicated that catechins, including catechin (C), EC, ECG, EGC, and EGCG, could inhibit the formation of AGEs *via* scavenging reactive oxygen species (44). Sang et al. found that EGCG efficiently trapped reactive dicarbonyl compounds (methylglyoxal and glyoxal) to prevent the formation of AGEs (45). Besides catechins, several flavonols and flavones also had anti-AGEs activity. Quercetin, kaempferol, apigenin, and luteolin were proved to inhibit the formation of AGEs by trapping reactive dicarbonyls (46). A further study revealed that the hydroxyl groups at the 3′-, 4′-, 5-, and 7-positions affected the inhibitory activity (47). Although the contents of catechins decreased, the contents of several flavonols and flavones were increased after baking (Figure 2). It might explain why the anti-AGEs activity was hardly reduced in the BSA + glyoxal reaction system.

The effects of baking on antibacterial activity are disadvantageous (Figure 3G). The MIC is the lowest concentration of a substance which prevents the visible growth of a bacterium or bacteria. A substance with low MICs is considered an effective antimicrobial agent. Compared with BT2, the MICs of BT0 and BT1 against *S. typhi* and *S. aureus* were lower, respectively. The MIC of BT0 against β-hemoly*tic Streptococcus* was lower than the MIC of BT1. The results indicated that the baking process attenuated the inhibition of TOTs against certain bacteria. Chou et al. proved that oolong tea had inhibitory effects on several micro-organisms (48). Monomeric polyphenols, which had a high affinity to proteins, were regarded as active antibacterial compounds (49). Some volatiles also displayed antibacterial activity, and there were synergistic interactions among specific volatiles. α-Pinene was capable of suppressing *S. aureus* (50). Indole, the second most abundant volatile in BT0, inhibited *Streptococcus mutans*. The combination of β-caryophyllene or δ-cadinene enhanced the inhibitory effects of indole against bacteria (51). The contents of monomeric catechins and the volatiles mentioned above (e.g., indole, α-pinene, and caryophyllene) were decreased after baking. The change was in accordance with the reduced antibacterial activity of BT1 and BT2. It implied that the loss of monomeric catechins and some volatiles during the baking process might be responsible for the baking-mediated decreased antibacterial activity of TOT.

## Conclusion

This study assessed and compared the chemical composition, sensory quality, and bioactivity of three TOTs with different baking degrees. According to the results, the baking process dramatically modified the chemical composition of TOTs, particularly in catechins, amino acids, and volatiles. The baking-induced changes of chemical components led to the darkened color of tea infusion, roast aroma, and enhanced sweet aftertaste. Since many flavor components are also bioactive components, the changes of chemical components also affected bioactivity. Adverse impacts of the baking process on the free radical scavenging activity and antibacterial activity were observed, probably due to the decrease of monomeric catechins. The results obtained herein fill the gap on the effects of the baking process of oolong tea and indicate that the baking process might be a double-edged sword, which enriches the flavor while attenuating the bioactivity. Future studies can focus on exploring the superior bioactivity of baked TOTs and developing processing technologies of TOTs that retain the typical sensory quality of baked TOTs but with less loss of bioactive components.

## Data Availability Statement

The raw data supporting the conclusions of this article will be made available by the authors, without undue reservation.

## Author Contributions

YG, J-FY, and Y-QX conceived and designed the experiments. YG, Q-QC, FW, J-XC, X-BZ, and Y-QX performed the experiments. YG, Q-QC, Y-HC, and J-QW analyzed the data. YG and DG wrote the manuscript. All authors have read and agreed to the final version of the manuscript.

## Conflict of Interest

The authors declare that the research was conducted in the absence of any commercial or financial relationships that could be construed as a potential conflict of interest.

## Publisher’s Note

All claims expressed in this article are solely those of the authors and do not necessarily represent those of their affiliated organizations, or those of the publisher, the editors and the reviewers. Any product that may be evaluated in this article, or claim that may be made by its manufacturer, is not guaranteed or endorsed by the publisher.
